# Endocranial Morphology in Metopism

**DOI:** 10.3390/biology14070835

**Published:** 2025-07-09

**Authors:** Silviya Nikolova, Diana Toneva, Gennady Agre

**Affiliations:** 1 Department of Anthropology and Anatomy, Institute of Experimental Morphology Pathology and Anthropology with Museum, Bulgarian Academy of Sciences, 1113 Sofia, Bulgaria; ditoneva@abv.bg; 2 Department of Linguistic Modelling and Knowledge Processing, Institute of Information and Communication Technologies, Bulgarian Academy of Sciences, 1113 Sofia, Bulgaria; agre@iinf.bas.bg

**Keywords:** digital morphometry, endocranial surface, machine learning, metopic suture, µCT imaging

## Abstract

Comparative investigations on homogenous cranial series have demonstrated that metopism is linked to a specific configuration of the cranial vault; however, there are no comparative data concerning the endocranial morphology in this condition. This study aimed to compare the endocranial space in metopic and control crania using morphometric analysis and machine learning algorithms. For this purpose, a series of 230 (184 control and 46 metopic) dry crania of contemporary adult Bulgarian males were scanned using an industrial µCT system. The 3D coordinates of 47 landmarks were collected on the endocranial surface. All possible measurements between the landmarks were calculated as Euclidean distances. The resultant 1081 measurements represented the initial dataset, which was reduced to smaller datasets applying different criteria. The derived datasets were used for learning a set of classification models by machine learning algorithms. The morphometric analysis showed that in the metopic crania some segments of the anterior and middle cranial fossae were significantly longer, and the landmark endobregma was significantly closer to the anterior and middle sections of the cranial base. The most accurate model, with a classification accuracy of 85%, was the Naive Bayes one learned on a dataset of 69 attributes assembled after an attribute selection procedure.

## Simple Summary

Metopism is a condition in which the metopic suture, which usually closes within the first postnatal year, persists in adult individuals, and the frontal bone is bipartite. Comparative investigations have demonstrated that metopism is associated with a specific configuration of the cranial vault, a delayed closure of the major sutures, a presence of supernumerary bones, and an underdevelopment of the frontal sinus. However, there are no comparative data concerning the endocranial morphology in this condition. In this study, we aimed to test the hypothesis that in metopism the differences in the vault are also related to differences in the endocranial morphology. For this purpose, we compared the metopic and control series using morphometric analysis and machine learning approaches. The obtained results showed that in metopism the anterior sections of the cranial base as well as the vault were specific, and this finding confirmed our hypothesis. However, the peculiarities of the base and vault were expressed in different directions. This discrepancy showed that besides the closely interrelated development of the cranial base and vault, in metopism the morphology of the vault did not entirely reflect the morphology of the base and vice versa.

## 1. Introduction

Metopism is a condition in which the metopic suture, which usually closes within the first postnatal year [1], persists in adult individuals, and the frontal bone is bipartite. Although many hypotheses have been suggested [2,3,4], the etiology of metopism still remains unclear. Metopism has been considered an innocuous variation found of varying frequency in different past and contemporary populations [5,6]. Since the first observations on metopism, it has been established that metopic crania have distinct morphology, and the differences concern mainly, but only, the frontal bone [2,7,8]. Recently, comparative investigations on homogenous cranial series have given significant evidence that metopism is associated with a specific configuration of the nasofrontal region and the cranial vault [9,10,11], a delayed closure of the major sutures [12], a presence of supernumerary bones [9,11], and an underdevelopment of the frontal sinus [13]. These features are usually related to a generalized disturbance in the intramembranous ossification and are common findings overexpressed in some types of skeletal dysplasia [14]. Therefore, metopism has been considered a manifestation of ossification disturbance [3], which could vary from a combination of “innocuous” variations to skeletal dysplasia [10].

Although it has been established that the configuration of the cranial vault in metopism is specific [9,10,11], no differences have been found in the angle of the cranial base [15]. Aside from these findings, as far as we know, there are no comparative data about the endocranial space in metopism. It has been speculated that metopic crania have a slightly greater capacity [2], but this assertion has not been investigated further. The aim of this study was to test the hypothesis that in metopism, the differences in the vault are also related to differences in the endocranial morphology. For this purpose, a digital morphometry on the endocranial space in metopic and control cranial series was performed. The obtained data were analyzed in two steps: a comparative morphometric analysis using classical statistical methods and an evaluation of the classification performance of machine learning (ML) models as a measure of how well the metopic and control series could be distinguished based on the endocranial metric characteristics. This approach was adopted due to some common problems in the morphometric data concerning the sample (size and imbalance between the groups), distribution of the raw data, etc., limiting the choice of the appropriate statistical analyses. On the other hand, ML algorithms tend to be relatively more robust and are designed to learn from data to gain knowledge and to make classifications and predictions. However, most of the ML algorithms are affected by irrelevant attributes in the initial training datasets. Therefore, learning is commonly preceded by an attribute selection procedure aiming at eliminating the most irrelevant attributes and finding the best subset that yields the minimum classification error. In this study, the implementation of an attribute selection scheme enabled an objective selection of the most differing measurements of the endocranial space between the metopic and control series.

## 2. Material and Methods

### 2.1. Material

The study was conducted on dry crania of adult male individuals who participated and died in the wars at the beginning of the 20th century. The bone remains were hosted in the Bulgarian Military Mausoleum with Ossuary at the National Museum of Military History. The sample included a total of 230 crania distributed into a control (n = 184) and a metopic (n = 46) series. The study was approved by the Human Research Ethics Committee of the Institute of Experimental Morphology, Pathology and Anthropology with Museum at the Bulgarian Academy of Sciences (Protocol No 7/30.10.2018).

### 2.2. Methods

#### 2.2.1. Data Collection

The 3D volumetric images of the crania were generated using an industrial µCT system, Nikon XT H 225 (Nikon Metrology, Tokyo, Japan), under the following conditions: a voltage of 100 kV, a power of 10 W, a tube current of 100 µA, and an exposure time of 500 ms. The crania were rotated at 360°, and a series of 2000 sequential projections was acquired; CT Pro 3D (Nikon Metrology, Japan) was used for the image reconstruction. The image postprocessing was performed according to a previously elaborated protocol for image stack merging [16]. The voxels were isotropic; the spatial resolution was 97.549 μm.

**Figure 1:**
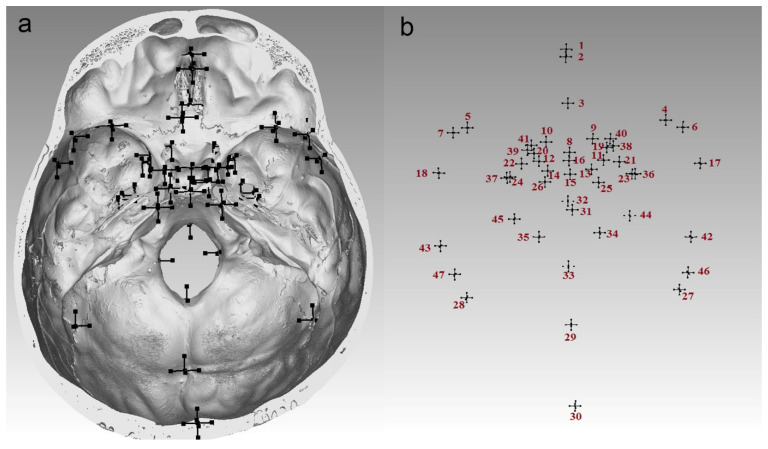
Landmark collection: (**a**) endocranial surface with picked landmarks; (**b**) landmarks in the order of their collection. The order is as follows: 1—foramen cecum (fc); 2—crista galli (cg); 3—crista galli base (cgb); 4—lesser wing of the sphenoid on the right (lwR); 5—lesser wing of the sphenoid on the left (lwL); 6—greater wing of the sphenoid on the right (gwR); 7—greater wing of the sphenoid on the left (gwL); 8— tuberculum sellae (ts); 9—optic canal on the right (ocR); 10—optic canal on the left (ocL); 11—anterior clinoid process on the right (acpR); 12—anterior clinoid process on the left (acpL); 13—posterior clinoid process on the right (pcpR); 14—posterior clinoid process on the left (pcpL); 15—dorsum sellae (ds); 16—sella (s); 17—endopterion on the right (eptR); 18—endopterion on the left (eptL); 19—foramen rotundum mediale on the right (frmR); 20—foramen rotundum mediale on the left (frmL); 21—foramen ovale mediale on the right (fomR); 22—foramen ovale mediale on the left (fomL); 23—foramen ovale laterale on the right (folR); 24—foramen ovale laterale on the left (folL); 25—petrous apex on the right (paR); 26—petrous apex on the left (paL); 27—endoasterion on the right (eaR); 28—endoasterion on the left (eaL); 29—internal occipital protuberance (iop); 30—endolambda (el); 31—endobregma (eb); 32—basion (ba); 33—opisthion (o); 34—foramen magnum laterale on the right (fmlR); 35—foramen magnum laterale on the left (fmlL); 36—foramen spinosum on the right (fsR); 37—foramen spinosum on the left (fsL); 38—foramen rotundum laterale on the right (frlR); 39—foramen rotundum laterale on the left (frlL); 40—superior orbital fissure on the right (sofR); 41—superior orbital fissure on the left (sofL); 42—parietal notch on the right (pnR); 43—parietal notch on the left (pnL); 44—occipitomastoid suture on the right (omsR); 45—occipitomastoid suture on the left (omsL); 46—superior petrous margin on the right (spmR); 47—superior petrous margin on the left (spmL).

The 3D coordinates of 47 (11 midsagittal and 18 bilateral) landmarks were collected on the endocranial surface (Figure 1, Table 1) using the “Clipping box” and “Indicator” tools in VG Studio Max 2.2. All possible measurements between these landmarks, in a total of 1081, were calculated as Euclidean distances using PAST [17].

#### 2.2.2. Data Analyses

##### Intraobserver Measurement Error

All landmarks were collected 3 times on 30 randomly selected crania by one examiner. Based on the landmark coordinates picked at each trial, three sets of measurements were calculated and compared. The intraobserver measurement error was evaluated using the technical error of measurement (TEM) and relative technical error of measurement TEM (rTEM).

##### Descriptive and Test Statistics

Basic descriptive indicators such as the mean, median, standard deviation (SD), minimum, maximum, 25th and 75th percentiles of the measurements were calculated for the metopic and control series. The significance of the differences in the measurements between the series was assessed by the independent samples *t*-test or the Mann–Whitney U-test depending on the normality and homoscedasticity of the data in the series, respectively. The level of significance was set at *p* ≤ 0.05.

##### Machine Learning

The experiments were conducted on a dataset in which the training examples were distributed into two classes: control crania (class 1) and metopic crania (class 2). The dataset consisted of 230 examples described by 1081 attributes (measurements); 184 examples belonged to class 1 and 46 to class 2. The classification accuracy of the default (majority-based—MB) classifier was 0.8 due to the considerable imbalance between the number of the samples in both classes.

Classifiers

Six effective ML algorithms [18] were selected for learning a set of classification models (classifiers) from the dataset: 1. CN2 [19]; 2. Logistic Regression (LR) [20]; 3. Multilayer Perceptron Neural Network (NN) [21]; 4. Support Vector Machines (SVM) [22]; 5. Naïve Bayes (NB) [23]; 6. Random Forest (RF) [24]. All algorithms were implemented in the Data Mining and Machine Learning environment Orange 2.7.8 (www.orangedatamining.com)

Classifier Evaluation Schema

The classification models created by the selected ML algorithms were evaluated by means of a repeated stratified k-cross-validation schema [21]. Due to the limitations related to our small sample size, instead of the commonly used 10-fold cross-validation schema, we applied a 5-fold cross-validation repeated 10 times at different randomly selected initial conditions. The quality of the models was evaluated by Average Classification Accuracy (Avr. CA), which counted a proportion of all correctly classified examples averaged across all 10 runs of the 5-fold cross-validation. The two class-dependent classification accuracies (Avr. CA 1 and Avr. CA 2) showed the proportion of correctly classified examples in each class, averaged across all 10 runs of the 5-fold cross validation. All classifiers were trained and tested on the same data subsets constructed by the 5-fold cross-validation in all 10 runs.

The selected classifiers were applied to the datasets assembled after an attribute selection procedure, as well as to a dataset consisting of all statistically significant attributes between the series.

Attribute Selection

Attribute selection was used to ignore the irrelevant and redundant attributes from the full dataset. Based on our previous experience with analogous datasets, we applied the following attribute selection procedure:

1. Attribute selection was performed using the Correlation-based Feature Selection method [25], implemented as the CfsSubsetEval algorithm in the Weka environment (https://www.cs.waikato.ac.nz/mL/weka/, accessed on 5 June 2025). Searching for the best subset of attributes was conducted via the Weka BestFirst algorithm [26];
2. The attribute selection method was tested in 5-fold cross-validation mode. Thus, the attribute selection was performed on 5 different subsets of the training sets containing 80% of the examples;
3. The previous step was repeated 10 times with randomly selected initial conditions. Thus, for each training dataset, 50 different subsets of selected attributes were learned from different subsets of the training datasets;
4. Attribute importance (AI) for a classification task was assessed based on the frequency of selection of the attributes;
5. According to the AI, values were assembled as subsets of attributes by intervals;
6. The selected ML algorithms were applied to the attribute subsets defined by the AI intervals in 10 × 5-fold cross-validation mode.

## 3. Results

### 3.1. Intraobserver Measurement Error

The intraobserver measurement error was evaluated using TEM and rTEM. The TEM values of all interlandmark distances were within 2 mm, and rTEMs were below the acceptable range of 5% (Supplementary Materials Table S1).

### 3.2. Morphometry

**Figure 2:**
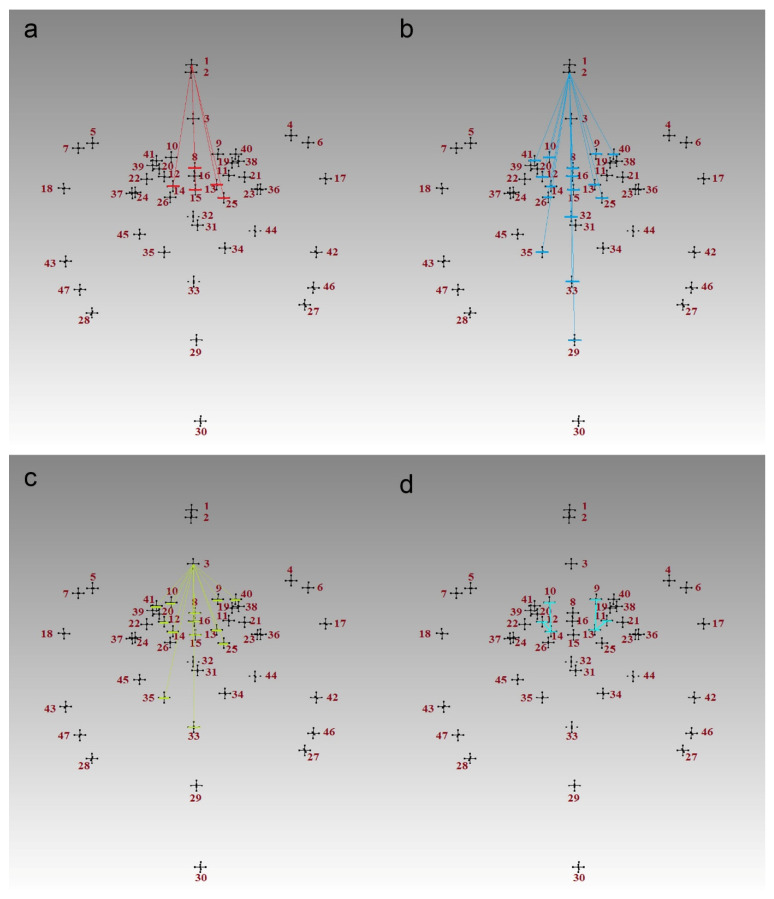
Significantly larger measurements in the metopic series: (**a**) from the landmark foramen cecum (1) designated with red lines; (**b**) from the landmark crista galli (2) designated with blue lines; (**c**) from the landmark crista galli base (3) designated with yellow lines; (**d**) on both sides between the landmarks on the optic canals (9, 10) and the posterior (13, 14) and anterior (11, 12) clinoid processes designated with light blue lines. The numbers correspond to the landmarks in the order of their collection (see Figure 1).

**Figure 3:**
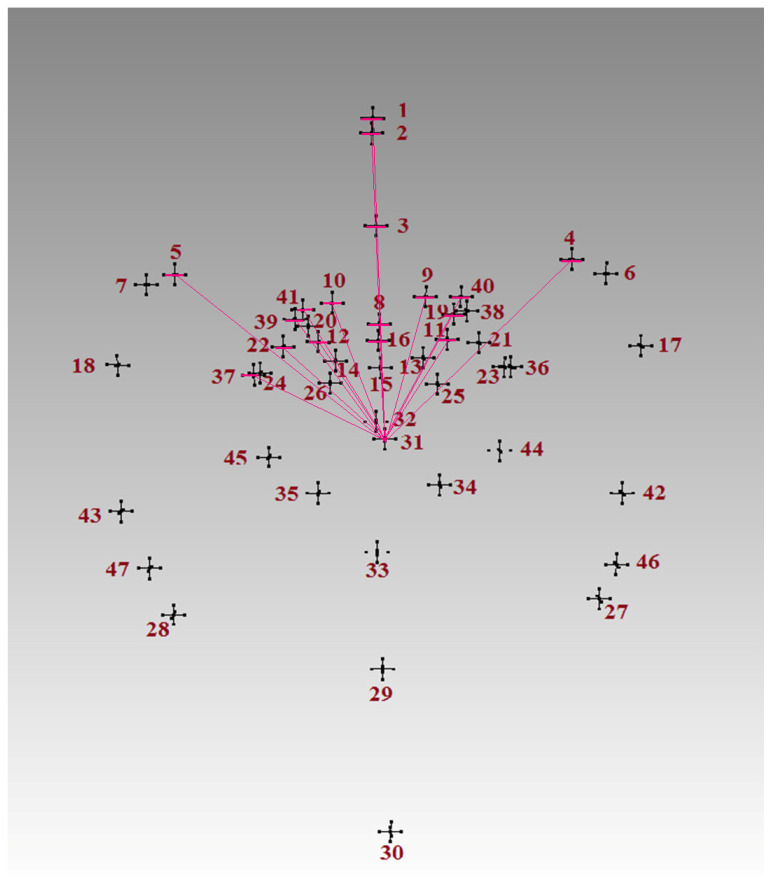
Significantly larger measurements in the control series starting from the landmark endobregma, designated with pink lines. The numbers correspond to the landmarks in the order of their collection (see Figure 1).

**Figure 4:**
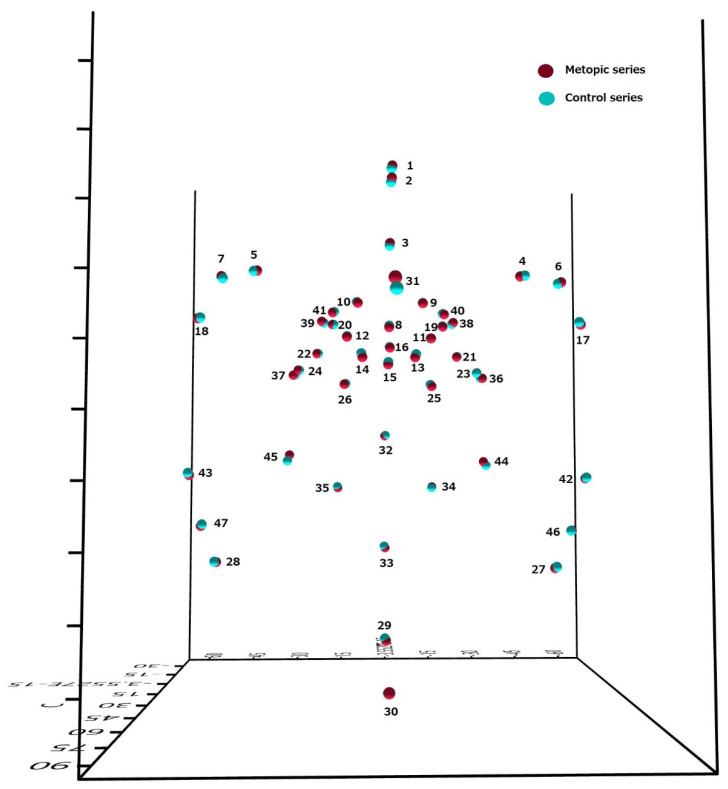
Visualization of the mean coordinates of the landmarks in the metopic (red spheres) and control (blue spheres) series in mm; the numbers correspond to the order of the landmark collection (see Figure 1).

The mean, standard deviation (SD), minimum and maximum values for all of the calculated measurements are presented as Supplementary Materials (Supplementary Materials Table S2). Out of the calculated 1081 measurements, one hundred and one were significantly different between the metopic and control series (Supplementary Materials Table S3). The distances between the landmarks at the anterior cranial fossa in the sagittal plane (fc, cg and cgb) and some landmarks surrounding sella turcica (op, ts, acp, pcl, ds and s) were significantly longer in the metopic series (Figure 2a–d). The distances from endobregma (eb) to foramen cecum (fc), crista galli (cg), crista galli base (cgb), lesser wings of the sphenoid (lwR and lwL), tuberculum sellae (ts), optic canals (opR and opL), anterior clinoid processes (acpR and acpL), sella (s), right foramen rotundum mediale (frmR), left foramen ovale mediale (fomL), left foramen spinosum (fsL), left foramen rotundum laterale (frlL), and superior orbital fissure (sofR and sofL) were significantly larger in the control crania (Figure 3). The mean coordinates of the landmarks in the metopic and control series are visualized in Figure 4.

### 3.3. Machine Learning

Attribute importance (AI) of the 69 selected attributes was given as a Supplementary File (Supplementary Materials Table S4). Accuracy of the selected classifiers learned on datasets constructed using an attribute selection procedure is presented in Table 2. The accuracy of the ML classifiers learned on the dataset of the significantly different 101 measurements is shown in Table 3. The CN2 classification rules with the best performance are presented in Table 4.

## 4. Discussion

Knowing the specific morphology of the skull in metopism will contribute to the detection of this condition not only when it is presented as a concomitant finding in genetic disorders related to impaired intramembranous ossification, but also when it is classified as an “innocuous variation”. In this study we calculated all possible distances between the collected landmarks on the endocranial space and obtained a full dataset, which we analyzed and reduced, extracting the significantly different measurements between the series and applying an attribute selection procedure. Then, we used the assembled datasets as input data for learning ML classifiers. Due to the applied approach, some of the resultant measurements did not comprise meaningful information about a particular structure but rather represented unilateral and/or oblique distances between unrelated structures.

Analysis of the morphometric data revealed that a total of 101 measurements were significantly different between the series. Some of these measurements were not related to a particular structure, i.e., they were without meaningful content for separation of the series. Considering the significantly different measurements that included one or two midsagittal landmarks, it could be seen that some distances between the structures in the anterior (fc, cg and cgb) and middle (sphenoid body) cranial fossae were significantly longer in the metopic crania. At the same time, in the control, the landmark endobregma was considerably more remotely placed from some of the structures in the anterior and middle cranial fossae. Although all of the points delineating crista galli (fc, cg, cgb) participated in significantly different measurements between the series, crista galli itself did not show any peculiarities in the metopic crania. It is worth noting that endolambda was not included in any significantly different meaningful measurement. Furthermore, there were no significantly different measurements between any of the opposite bilateral landmarks, i.e., none of the transverse diameters differed significantly between the metopic and control series.

Previous investigations revealed that in metopism there were significant differences in the cranial shape and size [9,10,11]. The main size modification was expressed in an enlargement of the frontal part of the neurocranium (enlarged transverse dimensions and shorter anteroposterior diameter) at the expense of the parietotemporal and occipital ones. The shape alteration in the metopic series represented a mediolateral widening and an anteroposterior shortening contributing to a globular outline of the cranium. Analysis of the endocranial morphology in this study revealed that some distances in the anterior and middle cranial fossae were longer in the metopic series, and the endobregma was situated closer to the anterior and middle segments of the cranial base. Moreover, none of the transverse diameters showed a significant difference between the series. Thus, in metopism the anterior part of the vault (frontal squama) was shorter in the anteroposterior direction, wider in the transverse plane, and more protruding in the midsagittal plane, whereas some segments of the anterior and middle cranial fossae were longer, there were no significant differences in the endocranial widths, and the endobregma was located closer to the cranial base. Apparently, in metopism the anterior sections of the cranial base as well as the vault were specific, and this finding confirmed the hypothesis, which we tested in this study. However, the peculiarities of the base and vault were expressed in different directions. This discrepancy showed that besides the closely interrelated development of the cranial base and vault, in metopism the morphology of the vault did not entirely reflect the morphology of the base and vice versa. It could be speculated that this specific morphology of the anterior segments of the cranial base and vault might be related to a specific morphology of the corresponding segments of the brain, but further investigations are needed.

The main purpose of the ML approach for data analysis was to extract the most distinctive characteristics between the metopic and control crania. The learned classification models were a way for evaluation of the classification ability of these features using different ML algorithms. In the first set of experiments, out of the full dataset of 1081 attributes, only 69 were selected. The AI was evaluated based on the selection frequency of each attribute during the 5-fold cross-validation mode repeated 10 times. Most of the selected attributes were of low importance (AI < 0.1, 58 in total). Eight attributes (fc-gwR; cg-pcpR; fc-eb; ba-omsL; cg-eptL; ocL-eb; ba-frlL; and fc-paR) were of low to moderate importance (AI between 0.1 and 0.4), and only three attributes (cgb-pcpR; cg-pcpL; and cgb-ts) were with AI > 0.4. It is worth noting that neither all of the selected 69 attributes (measurements) differed significantly between both series nor were all of them meaningful (Supplementary Materials Tables S3 and S4). However, the attributes with AI > 0.4 were significantly larger in the metopic series and characterized distances between structures in the anterior and middle cranial fossae (Figure 2). Due to the great imbalance between the number of the samples in both classes, the classification accuracy of the default (majority-based) classifier was 80.00 (100.00 for class 1 and 0.00 for class 2), and all used classifiers showed higher accuracy in classification of the examples in class 1 (Avr. CA 1 > 90%) compared to the examples in class 2 (Avr. CA 2 < 51%). Thus, the elaborated models classified the control crania, but they failed in classifying the metopic crania, and this could be related to the sample imbalance. The Avr. CA for both classes was between 80 and 85%, as the most accurate model was the Naive Bayes model (85%), while the CN2 rules (80%) were with the lowest classification accuracy (Table 2). Furthermore, the Avr. CA of the Naive Bayes, NN, SVM and LR models was higher when all of the initially selected 69 attributes were used, and any further selection based on the AI worsened the CA. The CA of the Random Forest model and CN2 rules was higher when they used only the attributes with AI > 0.04, which were 18 in total (Supplementary Materials Table S4).

The second set of experiments was conducted on a dataset consisting of all 101 significantly different measurements between the metopic and control series. Here, the classification accuracy of all learned models was lower compared to that of the models learned on datasets assembled after the attribute selection procedure. Once again, the most accurate classification ability was shown by the Naive Bayes (81%), SVM (80%) and NN (79%) models. The LR (77.9%) and CN2 (77.7%) showed similar and lower classification ability. All of the models classified the control series with higher accuracy (Table 3).

In both sets of experiments the most accurate classification models were learned by the subsymbolic algorithms: Naive Bayes, SVM and NN. The CN2 rules showed the lowest classification accuracy, but it is a symbolic and understandable for humans ML algorithm. However, the generated CN2 rules included some measurements between unrelated structures and did not reveal a clear tendency about the general differences between the metopic and control crania (Table 3). In general, the applied ML algorithms might be affected by the small number of the examples and the imbalance between the classes in the sample. In particular, the algorithms classified the control crania more accurately, but they failed in the classification of the metopic ones, which lowered the total classification ability of the models. Nevertheless, the attribute selection procedure was a useful step enhancing the classification accuracy and also an indicator for the most distinctive characteristic between the series.

## 5. Conclusions

The obtained results showed that in metopic crania some segments of the anterior and middle cranial fossae were significantly longer, and the landmark endobregma was significantly closer to the anterior and middle sections of the cranial base. Thus, in metopism similar to the vault, the endocranial morphology was also specific. However, the peculiarities of the vault and base were expressed in different directions. This discrepancy showed that in metopism the morphology of the vault did not entirely reflect the morphology of the base and vice versa.

The ML algorithms learned more accurate models using the dataset assembled after an attribute selection procedure compared to that learned on the dataset combining the significantly different measurements between the series. The most accurate model was learned by Naive Bayes (85%) on a dataset consisting of 69 attributes. This study contributes to the recognition of metopism and to the fundamental knowledge of skull morphology in this condition.

## Figures and Tables

**Table 1:** Description of the landmarks.

Landmark	Description
Midsagittal	
Basion (ba)	A point on the anterior margin of the foramen magnum in the midsagittal plane
Crista galli (cg)	The most prominent point on the crista galli
Dorsum sellae (ds)	A point at the dorsum sellae in the midsagittal plane
Endobregma (eb)	The point of intersection of the coronal and sagittal sutures on the endocranial surface
Endolambda (el)	The point of intersection of the sagittal and lambdoid sutures on the endocranial surface
Crista galli base (cgb)	A point placed at the posterior ridge of the crista galli in its base
Foramen cecum (fc)	A point on the posterior margin of the foramen cecum in the midsagittal plane
Internal occipital protuberance (iop)	A point on the internal occipital protuberance
Opisthion (o)	A point on the posterior margin of the foramen magnum in the midsagittal plane
Sella (s)	A point at the center of the sella turcica in the midsagittal plane
Tuberculum sellae (ts)	A point on the tuberculum sellae in the midsagittal plane
Bilateral	
Anterior clinoid process (acp)	The most prominent point on the anterior clinoid process
Endoasterion (ea)	The point of intersection of the lambdoid, occipitomastoid and parietomastoid sutures on the endocranial surface
Endopterion (ept)	The meeting point between the greater wing of the sphenoid, the parietal bone and the temporal squama on the endocranial surface
Foramen magnum laterale (fml)	The most lateral point on the margin of the foramen magnum
Foramen ovale mediale (fom)	The most medial point on the foramen ovale on the endocranial surface
Foramen ovale laterale (fol)	The most lateral point on the foramen ovale on the endocranial surface
Foramen rotundum mediale (frm)	A point on the medial ridge of the foramen rotundum
Foramen rotundum laterale (frl)	A point on the lateral ridge of the foramen rotundum
Foramen spinosum (fs)	A point on the foramen spinosum margin on the endocranial surface
Greater wing of the sphenoid (gw)	The meeting point between the greater wing of the sphenoid, the parietal bone and the frontal bone on the endocranial surface
Lesser wing of the sphenoid (lw)	The sharpest point at the site of the articulation with the frontal bone
Occipitomastoid suture (oms)	A point at the occipitomastoid suture, posterolateral to the jugular foramen on the endocranial surface
Optic canal (oc)	A point on the medial ridge of the optic canal
Posterior clinoid process (pcp)	The most prominent point on the posterior clinoid process
Parietal notch (pn)	The point of intersection of the squamous suture and the parietomastoid suture on the endocranial surface
Petrous apex (pa)	A point at the apex of the petrous part of the temporal bone
Superior orbital fissure (sof)	The most inferior point on the superior orbital fissure on the endocranial surface
Superior petrous margin (spm)	A point at the intersection of the superior margin of the petrous part of the temporal bone, formed between the anterior and posterior surfaces, with the parietomastoid suture, on the endocranial surface

**Table 2:** Accuracy of the best classifiers measured by 10 × 5-fold cross-validation.

Algorithm	CA	Class 1	Class 2
Majority	80.00	100.00	0.00
Naive Bayes 69 attributes with AI > 0	85.31 ± 1.12	94.00 ± 1.44	50.44 ± 3.52
Neural Network 69 attributes with AI > 0	82.35 ± 2.06	91.72 ± 1.52	50.36 ± 5.35
SVM 69 attributes with AI > 0	83.09 ± 0.97	98.27 ± 0.50	22.38 ± 4.49
Logistic regression 69 attributes with AI > 0	81.87 ± 1.93	90.42 ± 1.83	47.61 ± 5.26
Random Forest 18 attributes with AI > 0.04	81.48 ± 1.05	95.92 ± 0.82	23.69 ± 3.30
CN2 Rules 18 attributes with AI > 0.04	79.87 ± 1.74	93.77 ± 1.92	24.37 ± 5.86

**Table 3:** Accuracy of the models learnt from the 101 significantly different attributes validated using 10 × 5-fold cross-validation.

Algorithm	CA	Class 1	Class 2
Majority	80.00	100.00	0.00
Naive Bayes	81.35 ± 1.49	90.82 ± 1.60	43.72 ± 4.75
Neural Network	79.23 ± 1.65	90.05 ± 1.00	35.86 ± 5.86
SVM	80.31 ± 1.12	97.61 ± 1.04	9.57 ± 2.78
Logistic regression	77.87 ± 1.14	88.75 ± 1.17	34.35 ± 4.10
Random Forest	78.74 ± 0.66	94.24 ± 0.61	16.74 ± 2.18
CN2 Rules	77.65 ± 1.70	93.44 ± 1.93	13.70 ± 2.93

**Table 4:** The best CN2 classification rules predicting class 1 (control) and 2 (metopic series).

Rule Length	Rule Quality	Predicted Class	Distribution * Class 1:Class 2	Rule
3	0.98	1	62:0	IF 13-6 ≤ 46.0 AND 31-10 > 85.0 AND 2-35 > 80.0 THEN Class = 1
4	0.98	1	43:0	IF 8-3 ≤ 25.0 AND 31-10 > 86.0 AND 13-2 > 50.0 AND 12-2 ≤ 75.0 THEN Class = 1
3	0.96	1	26:0	IF 13-2 ≤ 52.0 AND 32-45 > 35.0 AND 23-44 ≤ 35.0 THEN Class = 1
2	0.94	1	16:0	IF 25-1 ≤ 65.0 AND 21-4 > 38.0 THEN Class = 1
2	0.93	1	12:0	IF 31-1 > 97.0 AND 31-1 ≤ 106.0 THEN Class = 1
3	0.93	2	0:12	IF 13-3 > 36.0 AND 21-4 ≤ 37.0 AND 8-3 > 25.0 THEN Class = 2
4	0.91	2	0:9	IF 10-41 > 12.0 AND 2-31 ≤ 85.0 AND 13-3 > 34.0 AND 25-1 > 66.0 THEN Class = 2
4	0.89	2	0:7	IF 2-1 ≤ 5.0 AND 13-6 > 50.0 AND 2-1 > 3.0 AND 13-6 ≤ 53.0 THEN Class = 2

* Number of the correctly classified examples within the classes.

## Data Availability

The data presented in this study are available on request from the corresponding author due to ethical reasons.

## Supplementary Materials

The following supporting information can be downloaded at https://www.mdpi.com/article/10.3390/biology14070835/s1, Table S1: Intraobserver measurement error; Table S2: Descriptive and test statistics of all measurements; Table S3: Descriptive and test statistics of the measurements with significant intergroup differences; Table S4: Attribute importance of the selected attributes.
